# Nicotinic Acetylcholine Receptors Modulate Bone Marrow-Derived Pro-Inflammatory Monocyte Production and Survival

**DOI:** 10.1371/journal.pone.0150230

**Published:** 2016-02-29

**Authors:** Stéphanie St-Pierre, Wei Jiang, Patrick Roy, Camille Champigny, Éric LeBlanc, Barbara J. Morley, Junwei Hao, Alain R. Simard

**Affiliations:** 1 Département de Chimie et Biochimie, Université de Moncton, Moncton, NB, Canada; 2 Department of Neurology and Tianjin Neurological Institute, Tianjin Medical University General Hospital, Tianjin, China; 3 Centre de Formation Médicale du Nouveau-Brunswick, Moncton, NB, Canada; 4 Boys Town National Research Hospital, Omaha, NE, United States of America; H. Lee Moffitt Cancer Center & Research Institute, UNITED STATES

## Abstract

It is increasingly clear that nicotinic acetylcholine receptors (nAChRs) are involved in immune regulation, and that their activation can protect against inflammatory diseases. Previous data have shown that nicotine diminishes the numbers of peripheral monocytes and macrophages, especially those of the pro-inflammatory phenotype. The goal of the present study was to determine if nicotine modulates the production of bone marrow -derived monocytes/macrophages. In this study, we first found that murine bone marrow cells express multiple nAChR subunits, and that the α7 and α9 nAChRs most predominant subtypes found in immune cells and their precursors. Using primary cultures of murine bone marrow cells, we then determined the effect of nicotine on monocyte colony-stimulating factor and interferon gamma (IFNγ)-induced monocyte production. We found that nicotine lowered the overall number of monocytes, and more specifically, inhibited the IFNγ-induced increase in pro-inflammatory monocytes by reducing cell proliferation and viability. These data suggested that nicotine diminishes the ratio of pro-inflammatory versus anti-inflammatory monocyte produced in the bone marrow. We thus confirmed this hypothesis by measuring cytokine expression, where we found that nicotine inhibited the production of the pro-inflammatory cytokines TNFα, IL-1β and IL-12, while stimulating the secretion of IL-10, an anti-inflammatory cytokine. Finally, nicotine also reduced the number of pro-inflammatory monocytes in the bone marrow of LPS-challenged mice. Overall, our data demonstrate that both α7 and α9 nAChRs are involved in the regulation of pro-inflammatory M1 monocyte numbers.

## Introduction

Cells of the monocytic lineage, including monocytes, macrophages and dendritic cells, are vital for the immune response and are involved in a multitude of inflammatory disorders [1–3]. Although all monocytic lineage cells originate from the same hematopoietic progenitors located in the bone marrow, the heterogeneity of their phenotype and their response to various stimuli is thought to explain the functional spectrum of these cells. Indeed, monocytic cell-based immune responses can be detrimental by causing local tissue damage, or beneficial by promoting tissue repair [1,4,5]. Two major subsets of monocytes and macrophages have been identified to date [6,7]. The first subset is often referred to as classically-activated monocytes/macrophages, pro-inflammatory monocyte/macrophages, or M1 monocytes, and their differentiation can be induced by IFNγ [8]. The second subset is regularly termed alternatively-activated monocytes/macrophages, anti-inflammatory monocytes/macrophages, or M2 cells, and are stimulated by IL-4 [8]. Monocyte subsets can be identified by their expression of a number of surface markers, where it is generally accepted that M1 cells are CD11b^+^/Ly6G^-^/Ly6C^high^/CCR2^high^/CX3CR1^low^ while M2 cells are CD11b^+^/Ly6G^-^/Ly6C^low^/CCR2^low^/CX3CR1^high^ [6]. Finally, M1 cells secrete high levels of the pro-inflammatory cytokines TNFα, IL-1β, IL-6 and IL-12 while M2 cells secrete the anti-inflammatory cytokine IL-10 and TGF-β [9–11]. The differences in the cytokine secretion profile of the two subsets partly explains why M1 cells are often linked to inflammatory or autoimmune disorders, whereas M2 cells are considered beneficial by promoting immune resolution and disease recovery. As such, a better understanding of the endogenous mechanisms that modulate monocyte/macrophage phenotypes could lead to the development of new therapeutic avenues for the treatment of inflammatory disorders.

It is now well-established that nicotinic acetylcholine receptors (nAChRs) are involved in mechanisms of immune regulation (reviewed in [12]). For instance, nAChR ligands such as nicotine can protect mice against various inflammatory diseases, including rheumatoid arthritis [13,14], sepsis [15] and experimental autoimmune encephalomyelitis (EAE), a mouse model for multiple sclerosis [16–18]. These molecules exert their beneficial effects by inhibiting the inflammatory functions of leukocytes [15–17,19–22]. The established actions of nicotine on cells of the monocytic lineage include the inhibition of pro-inflammatory cytokine (TNFα, IL-1β, IL-6 and IL-12) secretion concomitant with the upregulation of anti-inflammatory cytokine (IL-10, TGF-β) secretion [16,23,24]. The expression of pro-inflammatory monocyte markers MHC-II, CD80 and CD86 is also reduced in the spleen and central nervous system monocytic cells of nicotine-treated EAE mice [16,17]. Taken together, these data suggest that nAChRs may play a role in the regulation of the balance between M1 and M2 cells in peripheral and central nervous system tissue. It is still unclear, however, if such modulation of monocytes occurs during hematopoiesis in the bone marrow or subsequent to their release in the periphery. nAChRs have been implicated in hematopoiesis [25–27], supporting the former hypothesis. Moreover, it remains to be determined if nicotine exerts these effects directly by acting on nAChRs expressed by non-neuronal cells, or indirectly via other neuron-dependent immune regulatory pathways.

In the present study, we assessed the effects of nicotine on monocyte/macrophage cell production and differentiation into M1 monocytes. We accomplished this with primary cultures of monocyte colony-stimulating factor (M-CSF) and interferon gamma (IFNγ)-treated bone marrow cells and with an in vivo lipopolysaccharide (LPS)-induced inflammation model. We demonstrate that bone marrow cells (BMCs) express multiple nAChRs, and that nicotine decreases bone marrow-derived myeloid cell numbers by diminishing their proliferation and viability. Moreover, our data show that nicotine inhibits the expression of multiple pro-inflammatory markers and the secretion of pro-inflammatory cytokines via α7 and α9 nAChRs expressed by BMCs. We therefore conclude that α7 and α9 nAChRs are involved in the production of bone marrow M1 monocytes.

## Materials and Methods

### 1. Animals

C57BL/6J WT and α7 nAChRs knock-out (KO) mice (B6.129S7-Chrna^tm1Bay^, stock number 003232) were purchased from The Jackson Laboratory (Bar Harbor, MA, USA). The α9 nAChR KO heterozygous breeder mice, generated by the deletion of exons 1 and 2 containing the translation/transcription initiation sites, were generously provided by Barbara J. Morley, Boys Town National Research Hospital (Omaha, NE, USA). The α7 and α9 KO lines were maintained in a C57BL/6J background. The genotype of all lines was confirmed by PCR with the suppliers’ protocol and primer sequences: mouse nAChR α7 common forward primer 5’-TTC CTG GTC CTG CTG TGT TA-3’, α7 wild type (WT) reverse primer 5’-ATC AGA TGT TGC TGG CAT GA-3’ and α7 KO reverse primer 5’ -TAG CCG AAT AGC CTC TCC AC-3’ and mouse nAChR α9 WT forward primer 5’-GCC CCA TCC CTG CAT CT-3’, α9 WT reverse primer 5’-GTA GCT TTG GAA TGA GTG GAT GAG C-3’, nAChR α9 KO forward primer 5’-CGG ACC AAC TAA TGA TAC ACT GGA G-3’ and α9 KO reverse primer 5’-GAC CCA CAG AAT GAA CTG AGT TGA C-3’. Mice were housed in individually micro-ventilated cages, up to 5 mice per cage. Mice used were at least 7–8 weeks of age at the experiment's inception. Animals were anesthesized with 2–3% isoflurane, and immediately euthanized by cervical dislocation. All experiments involving animals performed in this study were reviewed, approved and conducted in accordance with the policies outlined by the Canadian Council for Animal Care and the Université de Moncton’s Animal Care Committee (Protocol number 13–02).

### 2. In vivo nicotine and LPS treatment

Nicotine Bitartrate (Sigma-Aldrich, Saint-Louis, MO, USA) was administered to mice as described previously [16]. Briefly, a 100 mg/mL solution in PBS or a solution of PBS alone was freshly prepared 24h before pump implantation and loaded into Alzet osmotic minipumps (model 1007D, Durect Corporation). The pumps were implanted subcutaneously on the right side of the back of the mouse while animals were kept under anesthesia using 2–3% of isoflurane, and continuously delivered either PBS or nicotine salt at ~13 mg of nicotine free base/kg/d for the duration of the experiment. After 4 days of nicotine treatment, LPS (1 mg/kg, Sigma-Aldrich) or an equivalent amount of PBS was injected in the intraperitoneal cavity. Mice were then euthanized 1, 6 or 24 hours after the LPS injection.

### 3. Tissue extraction and processing

Mice were placed under anesthesia using 2–3% isoflurane, USP (Partenaires Pharmaceutiques du Canada, Richmond Hill, ON, Canada). Blood was then quickly collected, followed by cervical dislocation to euthanize the mice. Afterwards, the spleen and bone marrow cells were obtained, as detailed below. *Blood extraction*: Between 0.5 mL and 1 mL of blood was obtained by cardiac puncture using 10% 0.5 M EDTA coated syringes and then transferred to an eppendorf tube containing 100 μL of 0.5 M EDTA. 300 μL of 3% dextran and 300 μL PBS was added to sediment red blood cells under room temperature for 45 min. The supernatant was obtained which contains mostly white blood cells. RBC lysis buffer (BioLegend, San Diego, CA, USA) was used to remove the rest of red blood cells, as per the supplied protocol. The single cell suspensions were then used for analysis by flow cytometry. *Spleen extraction*: Spleens were collected, and then gently dissociated by grinding on a 70 μm BD Falcon Cell Strainer and rinsed with PBS. Single cell suspensions were then treated with RBC lysis buffer to remove red blood cells, after which the single cell suspensions were ready for flow cytometry. *Bone marrow cell extraction*: Clean femoral and tibial bones from hind limbs were obtained by removing muscle tissues and sterilizing bones in EtOH 70%. BMCs were flushed from the bones with PBS using a 10 mL syringe and a 21-gauge needle for the femur, or a 25-gauge needle for the tibia, and collected in a 50 mL tube. Cell aggregates were dislodged by gentle pipetting. Red blood cells where lysed by RBC Lysis buffer, as per the supplier’s protocol. Debris was removed by passaging the suspension through a 40 μm BD Falcon Cell Strainer. Cells were harvested by centrifugation at 300 g for 10 minutes, and the supernatant was removed. Cells were then either used for quantitative real-time PCR, flow cytometry or culture, as described below.

### 4. Bone marrow cell cultures

BMCs were solubilized in complete medium (RPMI 1640 supplemented with 10% heat-inactivated FBS, 1% Pen/Strep, and 2 mM L-glutamine) at a concentration of 2X10^6^ cells/mL. For monocyte polarization experiments, CD11b^+^Ly6G^-^ monocytes were purified by fluorescence-activated cell sorting (FACS) as described in the flow cytometry section. Cells from each individual animal were then separated into three group of equal numbers of cells and plated in 6-well culture plates, 25 cm^2^ or 75 cm^2^ flasks. The first group was cultured either with complete RPMI 1650 or supplemented with 10 ng/mL recombinant human M-CSF (Sigma-Aldrich, Saint-Louis, MO, USA), as indicated. The second group was supplemented with M-CSF (10 ng/mL) and 2.5 ng/mL recombinant mouse IFNγ (Cell guidance systems, Cambridge, UK). The third group of cells was supplemented with M-CSF (10 ng/mL), IFNγ (2.5 ng/mL) and nicotine (100 μM) or an equivalent amount of PBS. The concentration of nicotine was chosen based on previously-published studies showing that the molecule has optimal anti-inflammatory effects in vitro at this concentration [16,17]. Cells were cultured in a humidified incubator at 37°C and 5% CO_2_. At the indicated time points (3 days for all experiments except the following: 24 hours for the polarization experiments and 4 days for the proliferation experiments), suspended cells were harvested by gentle pipetting of the medium, and adherent cells were washed with PBS and detached using a 10 mM EDTA solution at a pH of 7.4. Suspension and adherent cells were combined and spun at ~450 x g for 5 minutes. The supernatant was removed and cells were washed once by adding 10 mL of PBS followed by a 5 minute spin at 450 x g. After the wash, cells were counted and resuspended in PBS at a concentration of ≤1x10^6^ cells/100 μl. Cells were then used for flow cytometry.

### 5. RT-PCR

Fresh BMCs were used, in part, for RT-PCR. Cell were lyzed in 1 mL Ribozol (Amresco, Solon, OH, USA) for 1x10^7^ cells. As a positive control, the thymus (for α9 nAChR subunit expression) and the brain (for all other nAChR subunits) were also extracted from animals and homogenized in 1 mL Ribozol (Amresco, Solon, OH, USA) for 100 mg of tissue on ice with a glass homogenizer. Total RNA was purified using the Qiagen RNeasy mini Kit (Valencia, CA, USA) as per the supplied protocol. Total RNA yield and concentration was quantified using the NanoDrop 1000 (Thermo Scientific, Waltham, MA, USA). cDNA was then generated using the qScript XLT cDNA SuperMix (Quanta Biosciences, Gaithersburg, MD, USA) as per the supplied protocol, followed by RNA digestion with RNAse H (2U/rxn of 20 μL) at 37°C for 20 minutes (Ambion, Burlington, ON, CAN). Nested PCR were then performed on the T100 Thermal Cycler (BioRad, Hercules, CA, USA), using EconoTaq Plus Green 2X (Lucigen, Middleton, WI, USA) with template cDNA and the outer primers (Table 1). The second amplification was performed, with the same protocol, on amplified cDNA and the inner primers (Table 2). PCR product were run on a 2% agarose gel alongside negative control and PCR markers (Novagen, San Diego, CA, USA) stained with GelRed^™^ nucleic acid gel stain (Biotium, Hayward, CA, USA). Cycle conditions were: 95°C for 2 min, followed by 45 cycles of 95°C for 45 s, 60°C for 45 s and 72°C for 90 s and finally 72°C for 10 min.

**Table 1:** Outer primers sequence for nested PCR.

Gene (*mus musculus*)	RefSeq number	Forward primer sequence	Reverse primer sequence	Product size	Tm (°C)
α3	NM_145129.2	CTTGCCTGCCTGGGGTT	AGCACGATGTCTGGTTTCCA	508	60
α4	NM_015730.5	CTGCTTCCCTGACTGAGAGC	CAGATGGGTGTGTGCTGTCT	640	60
α6	NM_021369.2	CCCTTGCCCTTCACATCTGTT	TCTGCTGGAACTCGAAGTGT	534	60
α7	NM_007390.3	GGTCAAGAACTACAACCCGCT	CAAGGACCACCCTCCATAGG	436	60
α9	NM_001081104.1	GGGTGCTTTGTAGACAGAAGTG	TTGGTGATGGCTGGTGAGTC	522	60
β2	NM_009602.3	CAGGCACACTATTCTTCCGC	CAGACTTCAGTGGAGTGGGG	480	60
β3	NM_173212.3	AACTCCCGAGATGGCTTTGC	TCCTCCAGTCACAGAAGGCT	499	60
β4	NM_148944.4	TCCATTGTGGGGTGACCG	CAACACGATGTCAGGCAACC	384	60
HPRT	NM_013556.2	-	-	-	-
β2M	NM_009735.3	-	-	-	-

**Table 2:** Inner primers sequence nested PCR.

Gene (*mus musculus*)	RefSeq number	Forward primer sequence	Reverse primer sequence	Product size	Tm (°C)
α3	NM_145129.2	CTTAGCTGTGCTTCGGTGGT	AACAGGTACTGGAACAGGCG	176	60
α4	NM_015730.5	GGCAGTAGAAGGCGTCCAGT	GAGGCAGGAAGAGTCCCACA	164	60
α6	NM_021369.2	AGCCCTCCGTGTAGAAATCG	TGAAGCGGTTGTAGTGAGCA	185	60
α7	NM_007390.3	AGAACCAAGTTTTAACCACCAAC	CTGTTATAGAGGAGAATGTCTGGT	148	60
α9	NM_001081104.1	TACTCCAATGCTCTGCGTCC	CAGCCCGTCATACTGGTCTC	183	60
β2	NM_009602.3	GACGGTGTACGCTTCATTGC	TCGTGGCAGTGTAGTTCTGG	184	60
β3	NM_173212.3	CGGAGAGTAAGGGAACCGTG	GTCAAGAACCTGAGCCACGA	197	60
β4	NM_148944.4	TCCTCGTCTCTCTGTTCGCTC	GGATCAGGTTGTTGTAGCGG	111	60
HPRT	NM_013556.2	TGCTGACCTGCTGGATTACA	TTTATGTCCCCCGTTGACTGA	120	60
β2M	NM_009735.3	CTGGTCTTTCTGGTGCTTGTC	TATGTTCGGCTTCCCATTCTCC	108	60

### 6. Flow cytometry

#### 6.1 Phenotype analysis

Single cell suspensions of fresh BMCs extracted from non-treated, nicotine- or PBS-treated mice and the *ex vivo* treated cells were used at a concentration of ≤1X10^6^ cells/100 μL. Fc receptors were blocked by 1 μg/100 μL of anti-mouse TruStain fcX (Biolegend, San Diego, CA, USA) for 10 minutes at room temperature. Monocyte phenotype was analyzed by staining for one or more of the following mouse antigens (targeted by the indicated antibody fluorescently tagged with either Alexa Fluor 488, PE, APC or PE/Cy7): CD11b (clone M1/70), CD80 (16-10A1), CD86 (PO3), MHC-II (M5/114.15.2), CCR2 (clone 475301), Ly-6C (HK1.4) and Ly6G (1A8). All cytometry antibodies were purchased from Biolegend except for CCR2-PE, which was purchased from R&D systems (Burlington, ON, CAN). Appropriate isotype controls were always included. eFluor 670 (eBioscience, San Diego, CA, USA) was used to measure cell proliferation, while 7-AAD was used to assess cell viability, as per the supplied protocols. Samples were processed using a MoFlo XDP (Beckman Coulter, Pasadena, CA, USA) or a FC500 (Beckman Coulter). All cytometry results were analyzed using the Kaluza Software (Beckman Coulter).

#### 6.2 Cell sorting

Single cell suspensions of fresh BMCs extracted from non-treated mice were used to purify various BMC subpopulations. For hematopoietic stem cells (HSCs) and myeloid cell precursors, lineage-specific cells were first eliminated by magnetic cell sorting using the Mouse Hematopoietic Progenitor Cell Isolation kit (Stemcell Technologies, Vancouver, BC, Canada) as per the supplied protocol. Depleted cells were used at a concentration of under 1x10^6^ cells/100 μL. Fc receptors were blocked by adding 1 μg/100 μL of anti-mouse TruStain FcX (Biolegend) for 10 minutes at room temperature. Cells were then stained with anti-Sca-1 and anti-CD34 (Biolegend). HSCs were identified as Lin^-^Sca-1^+^CD34^-^ cells, while precursors were identified as Lin^-^Sca-1^-^CD34^+^ cells. For lineage-specific immune cell types, separate BMC extractions were used to sort monocytes (CD11b^+^Ly6G^-^), neutrophils (CD11b^+^Ly6G^+^) and B-lymphocytes (CD19^+^). Low FSC events were always discriminated against while sorting to eliminate cell debris and microparticles. All samples were processed using a MoFlo XDP. A small portion of sorted cells were analyzed using the FoFlo XDP to ensure the efficiency of the sorting.

### 7. ELISAs

BMCs stimulated by 10 ng/mL of M-CSF and 2.5 ng/mL IFN-γ were exposed to 100 μM nicotine or PBS for 4 days followed by an activation period of 6 or 18 hours by 10 ng/mL of LPS (Sigma Aldrich), with or without 100 μM nicotine. Supernatant was harvested by centrifugation at ~300 x g for 10 minutes and conserved at -80°C. The presence of the following cytokines in the supernatants was evaluated by ELISA using Peprotech’s (Rocky Hill, NJ, USA) ELISA Development kit: TNF-α, IL-1β, IL-12 and IL-10, following the manufacturer’s instructions. Fluorescence was quantified by a Varioskan (Thermo Electron Corporation, Marietta, OH, USA) and the results were analyzed by the SkanIt^™^ software by Thermo Scientific.

### 8. Statistical analysis

For all experiments with two factors, such as genotype and treatment, data were analyzed by two-way ANOVA. For experiments with treatment as the only factor, data were analyzed by one-way ANOVA. Due to the nature of our hypothesis and experimental design, all ex-vivo data were paired (repeated measures) per animal. In these paired experiments, differences between means were identified post-hoc with a Dunnett’s multiple comparisons test with the M-CSF + IFNγ group as the comparative control group. In one experiment for which we only had two groups to compare, a paired t-test was used to compare the means. For in vivo studies, a standard two-way ANOVA was used followed by Tukey’s post-hoc analysis. All statistical analyses were performed with GraphPad Prism 6 software. P-values of less than 0.05 were considered to be statistically significant. Results are always presented as means ± S.E.M.

## Results

### nAChRs are expressed by various bone marrow cell populations

It has previously been shown that binding sites for homomeric (α7, α9) and heteromeric (i.e. β2-containing) nAChR ligands are present in mouse BMCs, and that nAChR expression may be modulated throughout immune cell development [27,28]. However, the identity of specific nAChRs expressed by immune cells and their precursors remains unclear. We thus assessed nAChR mRNA expression in BMCs and in various BMC subpopulations, including HSCs, immune cell precursors, monocytes, neutrophils and B-lymphocytes. We first attempted to measure mRNA expression by qRT-PCR and found that nAChR mRNA levels in BMCs was near or below the detection threshold of 35 cycles for all nAChRs studied (data not shown). We thus performed nested PCR to detect the presence of nAChR mRNA in individual samples (Table 3). We found that nAChR mRNA expression in BMCs is highly heterogeneous and highly variable between samples, except for the α9 and β2 nAChR subunits, which were detected in nearly all samples (6 of 7 and 7 of 7, respectively). Surprisingly, α7 nAChR mRNA was found in 4 of 7 samples, while other subunits were expressed in 3 or fewer samples. To identify the source of this variation, we purified various BMC subpopulations by FACS. HSCs only had detectable levels of the β2 nAChR subunit, which was also found in all immune cell precursors and lineage cell samples. In addition, immune cell precursors had variable levels of α7 and α9 nAChR mRNAs. α9 nAChR expression was expressed in all lineage cells tested (monocytes, neutrophils and B-lymphocytes). β3 and β4 nAChR mRNAs were also observed in the majority of lineage cell samples, however, the α3, α4 and α6 nAChR subunits, which are the heteromeric binding partners for the β2, β3 and β4 subunits, were rarely expressed by these cells. Interestingly, the α7 nAChR subunit was found in 2 of 3 monocyte samples, 1 of 3 B-lymphocyte samples, and undetectable in neutrophils. Overall, these data demonstrate that the various BMC subpopulations express a variety of nAChRs and suggests that the expression of these receptors increases as immune cells progress in their development.

**Table 3:** nAChR mRNA expression in bone marrow cells.

# Positive samples	α3	α4	α6	α7	α9	β2	β3	β4
**Bone marrow**	3/7	2/7	2/7	4/7	6/7	7/7	3/7	1/6
**Stem cells**^§^	0/2	0/2	0/2	0/2	0/2	2/2	0/2	1/2
**CD34**^**+**^ **precursors**^§^	0/2	0/2	0/2	1/2	1/2	2/2	0/2	1/2
**Monocytes**	0/3	0/3	1/3	2/3	3/3	3/3	2/3	2/3
**Neutrophils**	0/3	0/3	1/3	0/3	3/3	3/3	3/3	3/3
**B cells**	0/3	0/3	0/3	1/3	3/3	3/3	1/3	3/3

Total RNA was extracted from bone marrow cells and the indicated FACS-purified bone marrow subpopulations of wild type mice. The expression of nAChRs was then assessed by nested RT-PCR. If a distinguishable band at the expected size was observed, the sample was considered to be positive for the expression of the tested nAChR. As depicted, transcripts of α7, α9 and β2 nAChRs were most frequently observed, whereas only a minority of samples expressed detectable levels of the other nAChRs tested. This suggests that some nAChRs are consistently expressed in bone marrow cells (α7, α9 and β2), while these cells are highly heterogeneous for other receptor subtypes. Of all the bone marrow cell subpopulations tested, only the β2 subunit was detected in hematopoietic stem cells, while other subunits were increasingly detectable as the cells differentiated into immune cells.

^**§**^ indicates that cells from 2 animals were pooled for each sample.

### Nicotine reduces BMDM cell numbers

**Fig 1:**
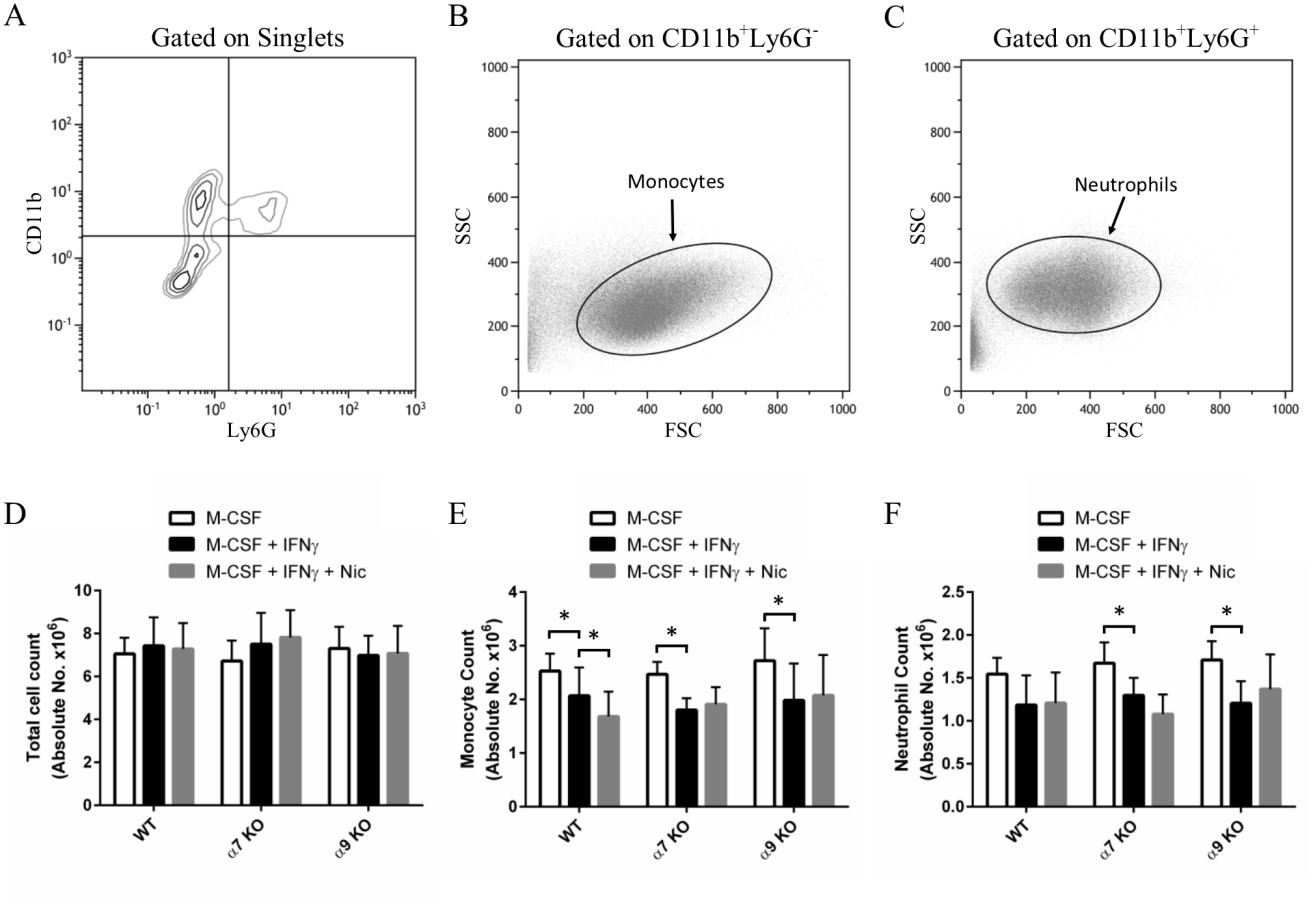
Monocyte cell numbers are reduced in WT mice following nicotine treatment ex vivo. Bone marrow cells were freshly isolated from WT, α7 KO and α9 KO mice and cultured for 3 days in the presence of M-CSF and IFNγ, with or without nicotine. Cells were then analyzed by flow cytometry, as follows (panels A-D): **A**, While gating on singlets, we identified CD11b^+^Ly6G^-^ and CD11b^+^Ly6G^+^ populations, which include monocytes and neutrophils, respectively. **B-C**, To eliminate cellular debris from the analysis, a new FSC vs SSC dot plot was generated while gating on CD11b^+^Ly6G^-^ cells (B) to identify monocytes or on CD11b^+^Ly6G^+^ cells (C) to identify neutrophils. The proportions (% Gated) of monocytes and neutrophils with respect to total cells (singlets) were thus obtained and used in the calculation of absolute cell counts. **D**, Total cell counts following the 3 days of cell culture were similar across all treatment groups and genotypes. **E**, Monocyte cell counts were obtained by multiplying the proportion of monocytes value with the total cell counts. Nicotine reduced monocyte numbers in WT mice only. **F**, Neutrophil cell counts were similarly obtained, and nicotine treatment had no effect. Data shown are means ± SEM (n = 4 per group), while * denotes P < 0.05 between indicated groups (two-way ANOVA for repeated measures, Dunnett’s post-hoc test).

Since we found that BMCs, especially monocytes, consistently express α9 nAChRs, and that α7 nAChRs are known for their role in the modulation of inflammation [12], we tested the ability of nicotine to modulate bone marrow-derived myeloid cell numbers via these two nAChR subunits. Although the β2 nAChR was also consistently detected in BMCs, the role of this subunit was not tested because must assemble with alpha-nAChR subunits other than α7 or α9 [29], which were rarely detected, to form a functional receptor. BMCs from WT, α7KO and α9KO mice were thus cultured for 3 days either with M-CSF, M-CSF + IFNγ or M-CSF + IFNγ + nicotine. The numbers of bone marrow-derived monocytes/macrophages (BMDMs) and neutrophils were then assessed by flow cytometry. First, doublets were eliminated by gating on singlets, then BMDMs and neutrophils were identified by gating on CD11b^+^Ly6G^-^ and CD11b^+^Ly6G^+^, respectively (Fig 1A). We then discriminated between cells and debris based on typical monocyte/macrophage (Fig 1B) and neutrophil (Fig 1D) SSC vs FSC characteristics, thus allowing us to ascertain the percent of total cells that were either monocytes/macrophages or neutrophils. We first found that the total number of cells in each dish was similar in all treatment groups and genetic backgrounds (Fig 1D). The absolute numbers of monocytes/macrophages and neutrophils were then calculated by multiplying each cell percentages with their respective total cell counts. We found that there were fewer BMDMs after 3 days of treatment with M-CSF and IFNγ, compared to M-CSF alone, in WT, α7KO and α9 KO cells. Interestingly, nicotine treatment further reduced BMDM numbers in WT cells only (Fig 1E). On the other hand, nicotine did not alter neutrophil numbers in any of the genetic backgrounds tested, while an IFNγ-induced decrease in their numbers only reached statistical significance in α7KO and α9KO cells (Fig 1F). Overall, these data suggest that nicotine can reduce BMDM cell numbers without affecting other cell types in general, and does so via the α7 and α9 nAChR subtypes.

### Nicotine reduces pro-inflammatory M1 monocyte proportions

**Fig 2:**
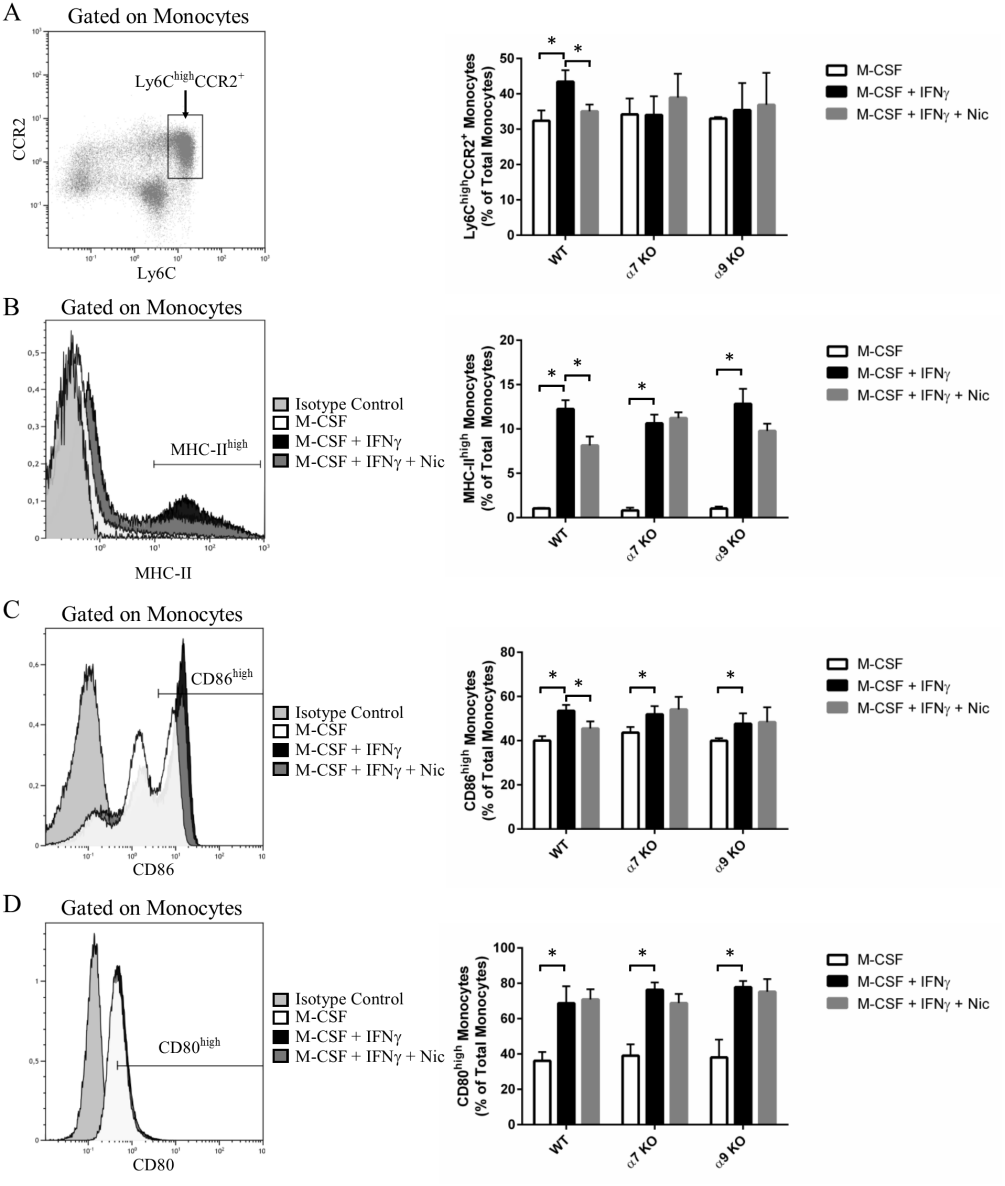
Nicotine decreases the proportions of proinflammatory monocytes. The expression of proinflammatory cell surface markers on monocytes was assessed by flow cytometry. Left panels are representative examples of dot plots or overlays used to assess marker expression, while group means are shown in graphical format on the right. **A**, the proportions of proinflammatory Ly6C^high^CCR2^+^ monocytes were reduced in WT mice following nicotine treatment. **B-D**, MHC-II (B) and CD86 (C) expression were inhibited by nicotine, while CD80 (D) expression levels were unaffected. Data shown are means ± SEM (n = 4 per group), while * denotes P < 0.05 between indicated groups (two-way ANOVA for repeated measures, Dunnett’s post-hoc test).

Multiple previous studies have shown that nicotine has general anti-inflammatory properties [15–17,19–22]. We therefore tested whether nicotine can alter the proportions of BMDMs that are pro-inflammatory in our cell culture model. While M-CSF induces the proliferation of all BMDMs, IFNγ induces their differentiation into the pro-inflammatory M1 phenotype [8], which express high levels of Ly6C and are also CCR2-positive [6]. We found that the proportions of Ly6C^high^CCR2^+^ BMDMs was higher in WT cell cultures treated with M-CSF and IFNγ compared to M-CSF alone, an effect that was completely prevented by nicotine in WT cells (Fig 2A). On the other hand, the IFNγ-induced increase in Ly6C^high^CCR2^+^ BMDMs was not observed, nor did nicotine have any additional effect, in α7KO or α9KO cells (Fig 2A). We also found that IFNγ induced the upregulation of the pro-inflammatory markers CD80, CD86 and MHC-II in monocytes in WT, α7KO and α9KO cells (Fig 2B–2D). Nicotine significantly inhibited the increase in MHC-II (Fig 2B) and CD86 (Fig 2C) expression in WT, but not in α7 KO or α9 KO cells. However, CD80 expression was unaffected by nicotine treatment (Fig 2D). These data thus suggest that nicotine can reduce the IFNγ-induced increase in pro-inflammatory BMDM proportions by acting on α7 and α9 nAChRs expressed by BMCs.

### Nicotine decreases the viability and proliferation of pro-inflammatory BMDMs

**Fig 3:**
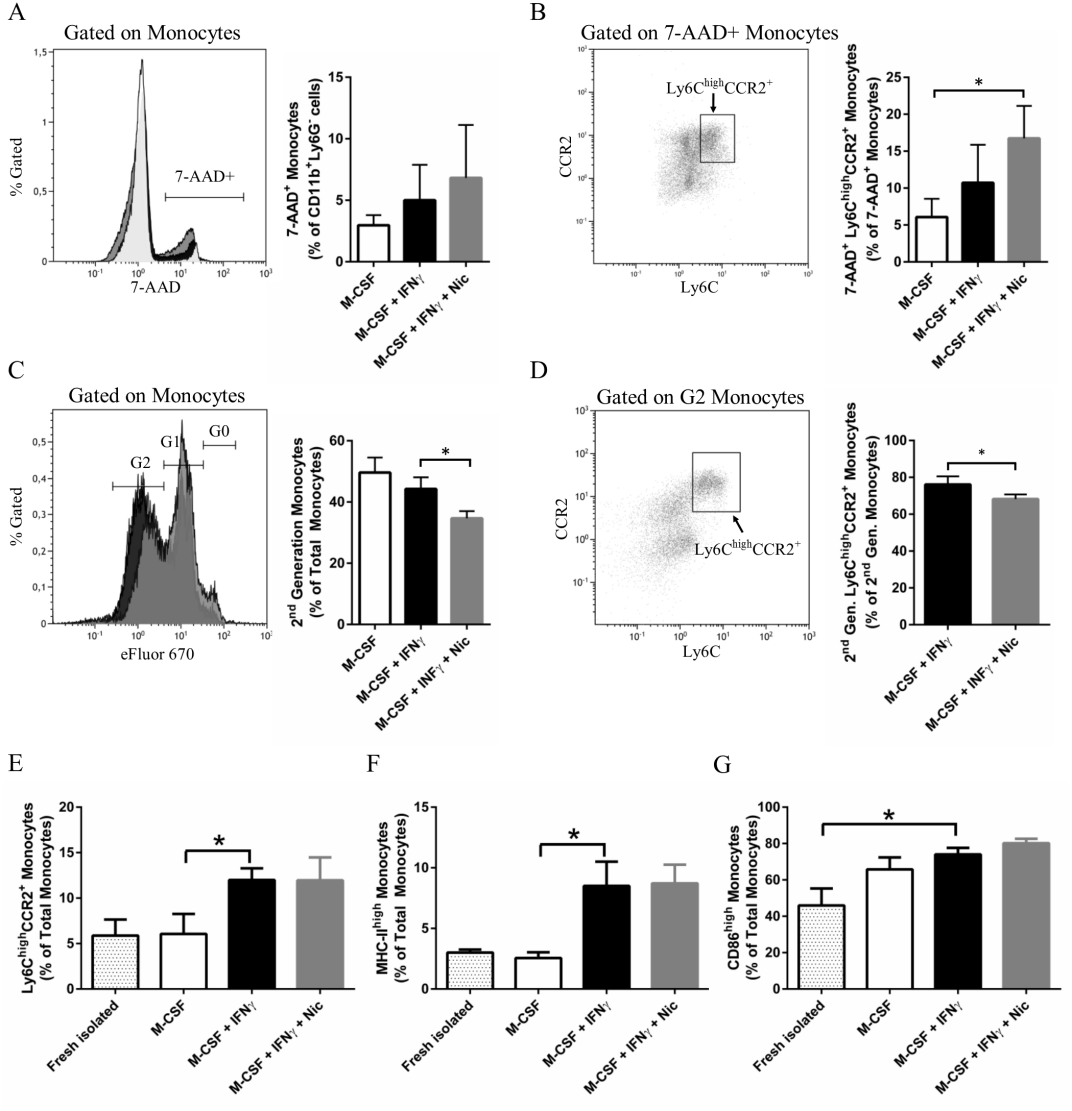
Nicotine decreases proinflammatory monocyte proportions by increasing cell death and decreasing cell proliferation. **A**, Cell viability was assessed in monocytes by flow cytometry with the use of 7-AAD after 3 days of culture. No statistically significant differences were found between groups. **B**, The proportions of 7-AAD^+^ monocytes that were Ly6C^high^CCR2^+^ was higher in nicotine-treated cells compared to the M-CSF group. **C**, in a separate experiment, cells were labelled with the Cell Proliferation Dye eFluor^®^ 670 prior to culture. After 4 days of culture, the number of monocytes that were of the 2nd generation (G2) increased following M-CSF + IFNγ treatment, an effect that was partially inhibited by nicotine. **D**, Similarly, the proportions of G2 monocytes that were proinflammatory was reduced by nicotine treatment. **E-G**, monocyte polarization in the pro-inflammatory phenotype was assessed in FACS-purified bone marrow monocytes. After 24 hours of culture, IFNγ induced pro-inflammatory monocyte polarization, as detected by increased proportions of monocytes that were Ly6C^high^CCR2^+^ (E), MHC-II^high^ (F) and CD86^high^ (G). This polarization was not inhibited by nicotine. Data shown are means ± SEM (n = 4 per group), while * denotes P < 0.05 between indicated groups (one-way ANOVA for repeated measures, Dunnett’s post-hoc test for all experiments except panel D, which was analyzed by a paired student’s t-test).

We next questioned whether nicotine can reduce pro-inflammatory BMDM proportions by inducing cell death, inhibiting their proliferation or preventing their polarization. We first assessed the effect of nicotine on monocyte viability in BMCs of WT mice by analyzing the percentage of monocytes that stained positive for the cell death marker 7-AAD (Fig 3A). Compared to cells treated with M-CSF + IFNγ, nicotine did not increased the percentage of 7-AAD^+^ monocytes. We then determined the percentage of 7-AAD^+^ monocytes that were Ly6C^high^CCR2^+^ (Fig 3B) and found that the proportion of M1 monocytes in the 7-AAD^+^ gate was increased in the nicotine-treated group compared to M-CSF treatment alone. In parallel (Fig 3C), we assessed the proliferation of monocytes and found that the percentage of 2^nd^ generation (G2) monocytes was lower in M-CSF + INFγ + Nic–treated cells compared to M-CSF and M-CSF + IFNγ –treated cells. As shown in Fig 3D, the percentage of G2 monocytes that were Ly6C^high^CCR2^+^ was also inhibited by nicotine. Finally, we tested whether nicotine could prevent the polarization of monocytes into the M1 phenotype by first purifying bone marrow monocytes of non-treated mice by FACS, and then culturing the cells for 24 hours in the presence of M-CSF, IFNγ and nicotine. Afterwards, we quantified the proportions of monocytes that were Ly6C^high^CCR2^+^ (Fig 3E), MHC-II^high^ (Fig 3F) or CD86^high^ (Fig 3G). These experiments revealed that nicotine was unable to inhibit the IFNγ-induced polarization of pro-inflammatory monocytes. Overall, these data suggest that nicotine reduces M1 monocyte proportions in BMCs ex vivo by increasing cell death and inhibiting their proliferation, but not by preventing their polarization.

### Nicotine inhibits the secretion of pro-inflammatory cytokines and promotes IL-10

**Fig 4:**
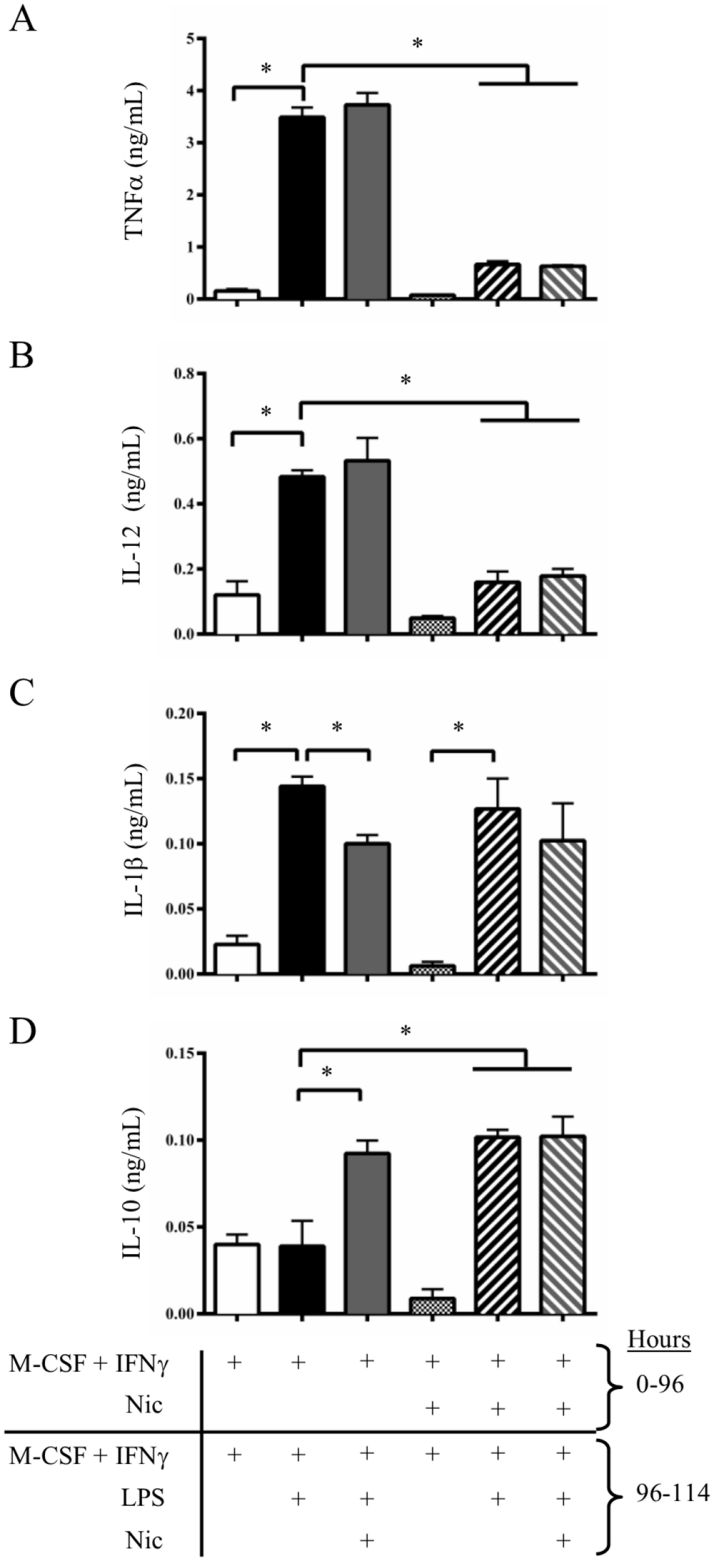
Nicotine treatment inhibits the IFNγ + LPS-induced pro-inflammatory cytokine secretion profile. Bone marrow cells were obtained and differentiated by M-CSF and IFNγ with or without nicotine for 4 days. Cells where then activated by LPS for 6 (TNFα) to 18 (IL-1β, IL-12 and IL-10) hours with or without nicotine. Afterwards, the supernatant was collected and cytokine concentrations were measured by sandwich ELISA. **A-B**, Nicotine treatment during the 4-day differentiation of BMDMs reduced the secretion of TNF-α (A), and IL-12 (B), two pro-inflammatory cytokines. **C**, the secretion of IL-1β, another pro-inflammatory cytokine, was also reduced by nicotine, but only when the drug was administered during the LPS activation stage. **D**, Nicotine treatment also increased IL-10 expression, regardless of the timing of treatment. Nicotine therefore inhibits the M1 pro-inflammatory cytokine secretion profile and induces an M2-like response. Data shown are means ± SEM (n = 4 per group), while * denotes P < 0.05 between indicated groups (one-way ANOVA for repeated measures, Dunnett’s post-hoc test).

To further confirm that nicotine reduced the production of pro-inflammatory monocytes, we quantified cytokine production by ELISA. In this experiment, we first cultured freshly-isolated BMCs in the presence of M-CSF + IFNγ with our without nicotine for 4 days. Cells were then collected, washed and re-seeded at equivalent concentrations, followed by another 6–18 hours of culture in the presence of M-CSF + IFNγ with or without nicotine and with or without LPS to stimulate cytokine secretion. Cytokines measured were TNFα after 6 hours (Fig 4A), while IL-12 (Fig 4B), IL-1β (Fig 4C) and IL-10 (Fig 4D) were quantified after 18 hours of stimulation. We found that nicotine treatment prevented TNFα and IL-12 secretion, but only when the drug was present during the first 4 days of culture. On the other hand, IL-1β secretion was also partially inhibited by nicotine, but only when nicotine was present during the LPS stimulation phase. Contrarily, IL-10 secretion was increased by nicotine, regardless of the timing of treatment.

### Nicotine inhibits M1 monocyte production in vivo

**Fig 5:**
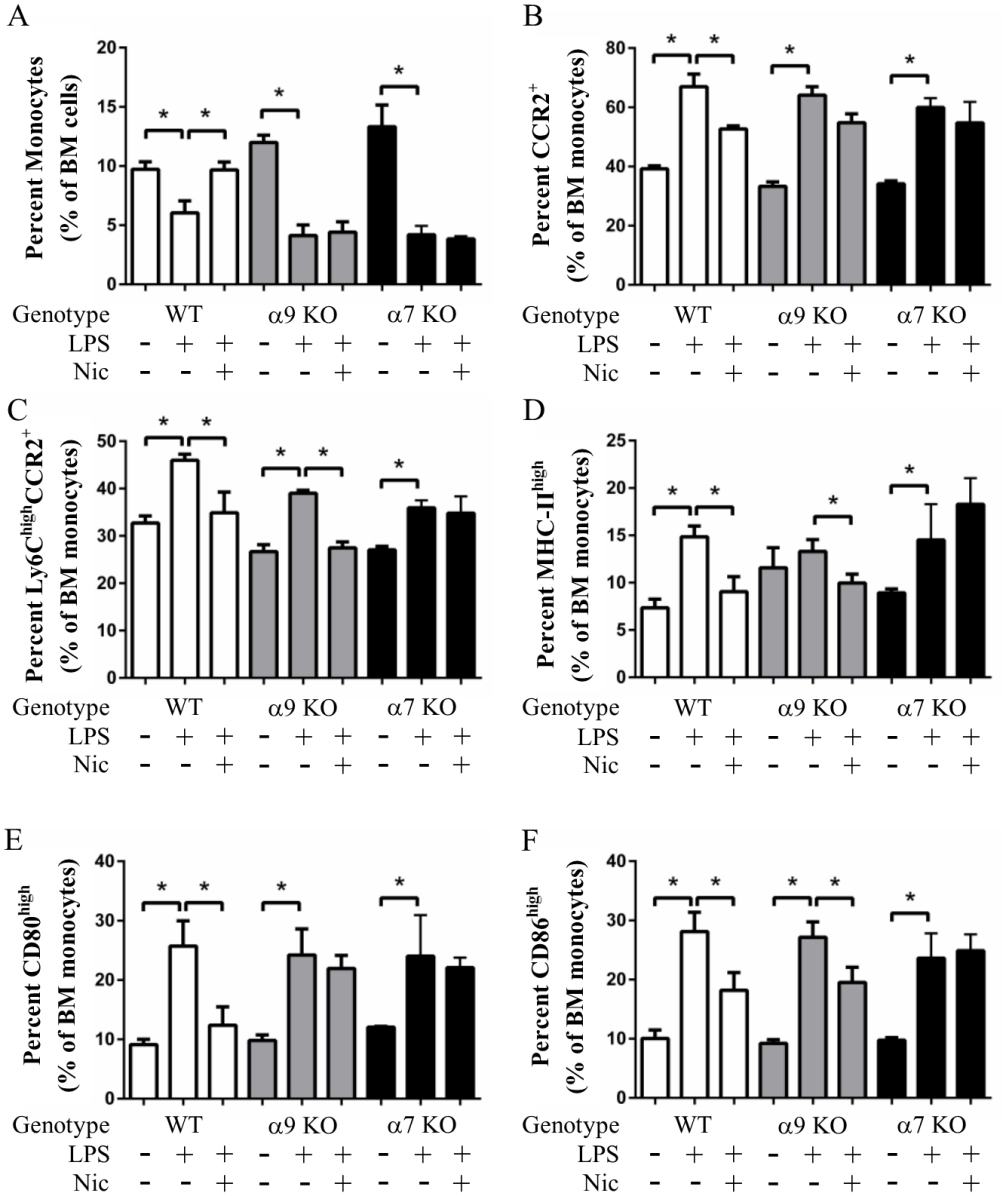
Nicotine modulates proinflammatory monocyte proportions and phenotype in the bone marrow in vivo. WT, α7 KO and α9 KO mice were treated with nicotine for 4 days and then injected with a single dose of LPS. Bone marrow was collected 24 hours later, and monocytes were analyzed by flow cytometry. **A**, Monocyte proportions were reduced by LPS treatment in the bone marrow, and effect that was prevented by nicotine in WT mice. **B**, The proportions of monocytes that expressed CCR2 was reduced in the bone marrow of nicotine-treated WT mice, an effect that was not observed in α7 KO and α9 KO mice. **C**, The proportions of Ly6C^high^CCR2^+^ cells were lower in the bone marrow of nicotine-treated WT mice and α9 KO mice, but not α7 KO mice. **D**, MHC-II^high^ monocyte proportions were also lower in the bone marrow of nicotine-treated WT and α9 KO mice, while nicotine’s effect was not present in α7KO mice. **E**, Nicotine reduced the proportions of monocytes that expressed high levels of CD80 in WT mice, but not in α9 KO or α7 KO mice. **F**, There were fewer CD86^high^ monocytes in nicotine-treated WT and α9 KO mice, but not in α7 KO mice. Overall, these data demonstrate that nicotine inhibits the presence of pro-inflammatory monocytes in the bone marrow of mice. Data shown are means ± SEM (n = 4 per group), while * denotes P < 0.05 between indicated groups (two-way ANOVA, Tukey’s post-hoc test).

We next evaluated if our ex vivo results could be replicated in vivo following 24 hours of LPS stimulation. WT, α7 KO and α9 KO mice were thus pre-treated for 4 days with nicotine via osmotic pumps implanted sub-cutaneously. A single intra-peritoneal dose of LPS was then administered and mice were sacrificed 24 hours later. Monocyte proportions in the bone marrow was then assessed by flow cytometry. We found that LPS reduced the numbers of monocytes in the bone marrow, an effect that was prevented by nicotine in WT, but not α7 KO or α9 KO mice (Fig 5A). Monocyte migration out of the bone marrow is regulated by CCL2, the ligand for CCR2 [30,31]. In order to explain the increased amounts of monocytes in the bone marrow and decreased levels pro-inflammatory monocytes in the blood of nicotine-treated mice, we determined the CCR2 surface expression levels of the general population of bone marrow monocytes (Fig 5B). Indeed, there was a significantly lower expression of CCR2+ monocytes in the bone marrow of nicotine-treated WT mice, but not in either KO mice. On the other hand, our data revealed that the percentage of M1 monocytes in the bone marrow was significantly increased by LPS, while this increase was prevented by nicotine treatment in WT mice (Fig 5C). Interestingly, nicotine retained its ability to reduce this cell population in α9 KO animals. Similarly, we also found that the proportions of MHC-II^high^ (Fig 5D), CD80^high^ (Fig 5E) and CD86^high^ (Fig 5F) monocytes in the bone marrow were increased by LPS stimulation, while this effect was prevented by nicotine in WT mice. Again, nicotine maintained its ability to inhibit MHC-II and CD86, but not CD80, expression in α9 KO mice, while the nAChR ligand’s effects were reversed in α7 KO mice. These data suggest that nicotine prevents the migration of monocytes out of the bone marrow by modulating CCR2 surface expression, and that the nAChR ligand lowers the ratio of pro-inflammatory monocyte production within this immune compartment.

## Discussion

Previous studies have shown that nAChRs can influence the general production of immune cells in the bone marrow of mice [25–27]. The data presented in this manuscript not only support these prior findings, but provide new evidence that nAChRs can modulate the number of pro-inflammatory monocytes in the bone marrow by inhibiting their proliferation and decreasing their viability. In addition, our results demonstrate that α7 nAChRs play a major role, and suggest that α9 nAChRs are also involved, in the regulation of pro-inflammatory monocyte numbers. Prior to the current study, it was also unclear whether nAChR ligands could exert their effects on bone marrow cells by acting directly on receptors expressed by these cells, or indirectly by modulating other systemic pathways involved in hematopoiesis. Our data support the notion that nAChRs in bone marrow cells, and especially those found in immune cells themselves, are important mediators of pro-inflammatory monocyte production. Finally, our findings lend further evidence that nAChRs play a role in the retention of monocytes in the bone marrow [32,33].

In this study, we first assessed nAChR mRNA expression in freshly isolated bone marrow and in FACS-purified bone marrow cell subpopulations. These included HSCs, immune cell precursors, as well as monocytes, neutrophils and B cells. Overall, we found that HSCs only express detectable levels of the β2 nAChR subunit, whereas other nAChR subunits were expressed in cells committed to the immune lineage. In fact, our data suggest that the types and consistency of nAChR subunits expressed increases in parallel with the developmental stage of immune cell differentiation. The only nAChRs consistently expressed by immune cells and their precursors were the α9 and β2 subunits. The α7 nAChR subunit, previous demonstrated to be expressed by a portion of bone marrow cells [27,34], was found to be variably expressed in monocytes and B cells, and was not detected in neutrophils. The variability observed in α7 nAChR expression between individual samples from litter mates are in agreement with previous findings [34]. Other subunits, such as the β3 and β4 nAChRs were also variably expressed in immune cells of the bone marrow. However, the β2–4 nAChR subunits only form functional nAChRs when assembled with the α2–6 nAChR subunits [29], thus it is unlikely that functional heteropentameric nAChRs are present in bone marrow cells. This is contrary to a previous study, which found heteropentameric binding sites in a limited number of bone marrow cells [27]. These opposing results may be attributed to the sensitivity of the approaches used in both studies. Nonetheless, both studies support the notion that heteropentameric nAChRs represent a relatively small portion of all nAChRs present in the bone marrow. Furthermore, it is increasingly clear that homopentameric α7 and α9 nAChRs are the predominant functional subtypes found in immune cells, and that these receptors appear to be modulated throughout immune cell development.

We thus assessed the effect of nicotine, a nAChR ligand that can bind to all receptor subtypes, on BMDM and neutrophil production ex vivo and in vivo using WT, α7 KO and α9 KO mice. Our results demonstrate that nicotine reduces the numbers of total BMDMs, but not neutrophils, ex vivo using cells of WT mice. This effect is explained by data demonstrating that nicotine reduces the viability and proliferation of pro-inflammatory monocytes. It is highly unlikely that nicotine’s effects ex vivo were due to a general toxicity of this molecule at the concentration used, since nicotine did not reduce total cell numbers in culture, and rather only affected pro-inflammatory cells. On the other hand, our in vivo experiments revealed that nicotine increased total monocyte proportions in the bone marrow of WT mice. This contradiction is most likely due to nicotine’s role in BMC retention [32], which is highly dependent on CCL2/CCR2 signaling [30,31]. Indeed, nicotine significantly reduced CCR2 expression in monocytes, thus supporting this hypothesis. More importantly, nicotine prevented the ex vivo IFNγ-induced increase in pro-inflammatory monocyte proportions, as evidenced by the proportions of monocytes that were Ly6C^high^CCR2^+^, MHC-II^high^ and CD86^high^ in nicotine-treated cells. Our in vivo experiments confirm these findings, although in this case nicotine also prevented the increase in CD80^high^ monocyte percentages. The effect of nicotine on CD80 expression in vivo but not ex vivo may be due to the different stimuli (LPS vs INFγ) or other environmental or systemic factors that are present in vivo. The shift towards a less inflammatory phenotype was also confirmed by the fact that nicotine significantly inhibited the pro-inflammatory cytokine expression profile and increased IL-10, an anti-inflammatory cytokine. These results support multiple previous studies showing a role of α7 nAChRs in regulating the phenotype, oxidative stress markers and cytokine expression profile of bone marrow-derived monocytes and macrophages [35–38]. Considering our findings and that nAChRs also appear to play a pro-survival role in alternatively-activated M2 macrophages, [39], the importance of cholinergic signaling in modulating the balance of M1 vs M2 monocytes/macrophages is thus becoming increasingly clear.

In both models, the nicotinic reduction in M1 monocyte proportions was impaired in α7 KO mice, while the absence of α9 nAChRs only prevented the effect of nicotine ex vivo. It is therefore clear that α7 nAChRs are involved in this pathway, as expected due to this nAChR subtype’s established role in nicotine’s general anti-inflammatory properties. On the other hand, the fact that α9 nAChR gene deletion prevented nicotine’s effects ex vivo, but not in vivo, suggests that this subtype is only involved in nicotine’s direct actions via α9 nAChR expression in BMCs. More importantly however, this finding implies that the α7 nAChR, which is present in the α9 KO mice, may also take part in systemic anti-inflammatory pathways that are modulated by nicotine. It will thus be important to consider the ability of nAChRs and their ligands to act locally versus systemically when assessing the role of the cholinergic system in immune modulation.

Overall, these data are in agreement with previous studies that demonstrated the ability of nicotine to influence pro-inflammatory marker expression in circulating immune cells [16,17] and to inhibit inflammation [19–22,24]. More importantly, our results demonstrate for the first time that nicotine is able to directly modulate pro-inflammatory monocytes by inhibiting their production and release from the bone marrow. In addition, we provide evidence that α7 and α9 nAChRs are involved in cholinergic modulation of pro-inflammatory monocytes. Our findings thus contribute towards our understanding of the mechanisms that regulate monocyte production. Due to the prominent role of these cells in a multitude of inflammatory disorders, our data provide valuable information that can be used towards the goal of developing new anti-inflammatory treatments.

## Abbreviations

BMC: bone marrow cell
BMDM: bone marrow-derived monocyte/macrophage
EAE: experimental autoimmune encephalomyelitis
FACS: fluorescence-activated cell sorting
G2: 2^nd^ generation
IFNγ: interferon gamma
IL: Interleukin
KO: knock out
LPS: lipopolysaccharide
M1: classically-activated monocytes/macrophages
M2: alternatively-activated monocytes/macrophages
M-CSF: monocyte colony-stimulating factor
nAChR: nicotinic acetylcholine receptor
TNFα: tumor necrosis factor alpha
WT: wild type
